# Extensive Core Microbiome in Drone-Captured Whale Blow Supports a Framework for Health Monitoring

**DOI:** 10.1128/mSystems.00119-17

**Published:** 2017-10-10

**Authors:** Amy Apprill, Carolyn A. Miller, Michael J. Moore, John W. Durban, Holly Fearnbach, Lance G. Barrett-Lennard

**Affiliations:** a Department of Marine Chemistry and Geochemistry, Woods Hole Oceanographic Institution, Woods Hole, Massachusetts, USA; b Biology Department, Woods Hole Oceanographic Institution, Woods Hole, Massachusetts, USA; c Marine Mammal and Turtle Division, Southwest Fisheries Science Center, National Marine Fisheries Service, NOAA, La Jolla, California, USA; d SR^3^ SeaLife Response, Rehabilitation, and Research, Mukilteo, Washington, USA; e Coastal Ocean Research Institute, Vancouver Aquarium, Vancouver, BC, Canada; f Zoology Department, University of British Columbia, Vancouver, BC, Canada; Northern Arizona University

**Keywords:** SSU rRNA gene, bacteria, drone, humpback whale, microbiome

## Abstract

The pulmonary system is a common site for bacterial infections in cetaceans, but very little is known about their respiratory microbiome. We used a small, unmanned hexacopter to collect exhaled breath condensate (blow) from two geographically distinct populations of apparently healthy humpback whales (*Megaptera novaeangliae*), sampled in the Massachusetts coastal waters off Cape Cod (*n* = 17) and coastal waters around Vancouver Island (*n* = 9). Bacterial and archaeal small-subunit rRNA genes were amplified and sequenced from blow samples, including many of sparse volume, as well as seawater and other controls, to characterize the associated microbial community. The blow microbiomes were distinct from the seawater microbiomes and included 25 phylogenetically diverse bacteria common to all sampled whales. This core assemblage comprised on average 36% of the microbiome, making it one of the more consistent animal microbiomes studied to date. The closest phylogenetic relatives of 20 of these core microbes were previously detected in marine mammals, suggesting that this core microbiome assemblage is specialized for marine mammals and may indicate a healthy, noninfected pulmonary system. Pathogen screening was conducted on the microbiomes at the genus level, which showed that all blow and few seawater microbiomes contained relatives of bacterial pathogens; no known cetacean respiratory pathogens were detected in the blow. Overall, the discovery of a shared large core microbiome in humpback whales is an important advancement for health and disease monitoring of this species and of other large whales.

**IMPORTANCE** The conservation and management of large whales rely in part upon health monitoring of individuals and populations, and methods generally necessitate invasive sampling. Here, we used a small, unmanned hexacopter drone to noninvasively fly above humpback whales from two populations, capture their exhaled breath (blow), and examine the associated microbiome. In the first extensive examination of the large-whale blow microbiome, we present surprising results about the discovery of a large core microbiome that was shared across individual whales from geographically separated populations in two ocean basins. We suggest that this core microbiome, in addition to other microbiome characteristics, could be a useful feature for health monitoring of large whales worldwide.

## Abstract (precis)

The conservation and management of large whales rely in part upon health monitoring of individuals and populations, and methods generally necessitate invasive sampling. Here, we used a small, unmanned hexacopter drone to noninvasively fly above humpback whales from two populations, capture their exhaled breath (blow), and examine the associated microbiome. In the first extensive examination of the large-whale blow microbiome, we present surprising results about the discovery of a large core microbiome that was shared across individual whales from geographically separated populations in two ocean basins. We suggest that this core microbiome, in addition to other microbiome characteristics, could be a useful feature for health monitoring of large whales worldwide.

## INTRODUCTION

A number of large whale populations are listed as endangered or critically endangered (1), and their conservation and management greatly depend on understanding the relationship between anthropogenic disturbances and health (2–5). The pulmonary system is a common site of cetacean infection and disease (6, 7), and yet cases are diagnosed mostly in live-stranded or dead whales (8) and rarely in the wild. In humans, exhaled breath is used to test for bacterial and fungal infections of the lower respiratory tract (9). Therefore, examining the microorganisms in the exhaled breath of whales, commonly referred to as blow, may serve as an important indicator of whale respiratory health, including the identification of potential bacterial, fungal, and viral respiratory pathogens.

Advances in aerial drone technology offer new opportunities for studying the health of whales remotely and noninvasively. For example, the exhaled breath of large whales can be sampled with a small aerial drone (10), given that whales exhale large volumes of air when they surface and usually breathe several times during a surfacing interval. The exhaled breath contains mucus and moisture that, when released into the comparably cooler external air, condense to form a visible mass of vapor, which can be collected. Often blow is collected by approaching the whale in a small boat and holding an ~7-m pole with a collection plate above the blow hole (11, 12), requiring a skilled team and presenting safety risks to both the researchers and the whale. However, as mentioned above, blow was successfully collected by flying a remotely operated drone through the visible mass of vapor, thus offering a less invasive and safer platform for blow collections (10).

Knowledge of respiratory-associated microorganisms in cetaceans is limited. Several studies examined the diversity of respiratory-associated bacteria in captive and wild bottlenose dolphins (*Tursiops truncatus*) and provided preliminary evidence that dolphins host a core group of bacteria associated with the respiratory system (13–15). A cultivation-based study of blow from killer whales (*Orcinus orca*) identified pathogenic and antibiotic-resistant bacteria and fungi (16), which may be presenting health risks to the whales. The only study of blow-associated microorganisms in baleen whales was conducted using taxonomic screening for specific bacteria, leaving a number of questions remaining about the broader diversity of the blow microbiome (10). Given this limited knowledge about the large-whale respiratory microbiome and the possible implications of using the microbiome for health and pathogen monitoring, a broader understanding of the large-whale blow microbiome is needed.

**FIG 1:**
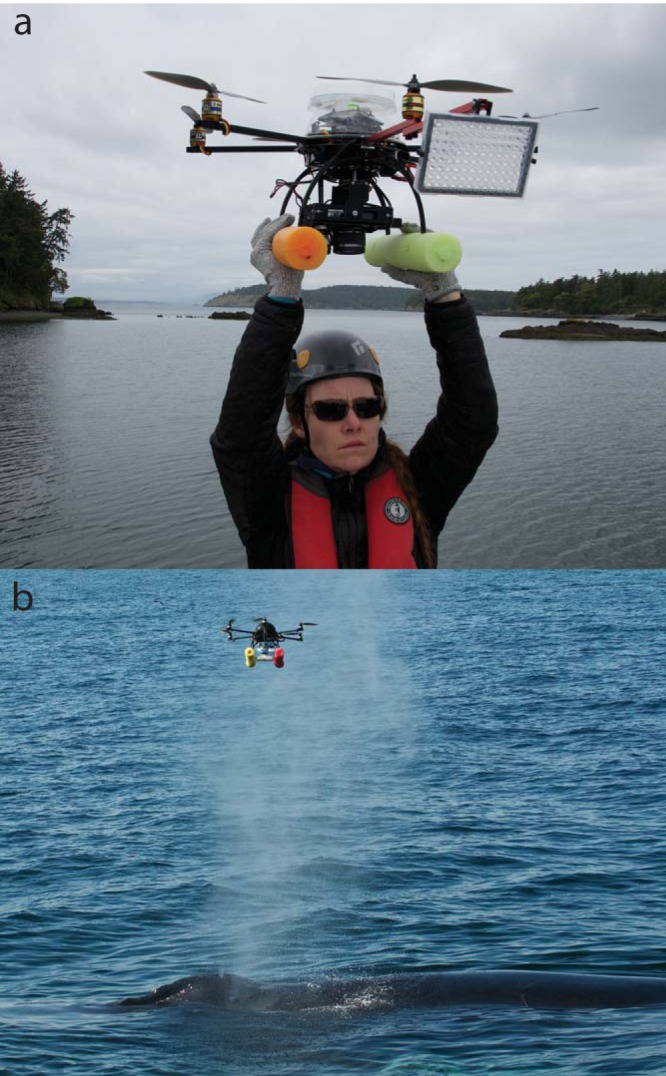
(a) Photograph of the APH-22 hexacopter launching for flight, with a petri dish atop and a 96-well PCR plate attached on a forward arm for whale blow sampling. (b) Photograph of the hexacopter collecting blow from a humpback whale off Cape Cod. Photographs courtesy of the authors.

In this study, we sought to determine if drone-captured blow microbiomes of large whales could be used to remotely monitor the respiratory health of large whales. Specifically, we sought to characterize the microbiomes associated with drone-collected samples of blow to assess commonalities and differences between the blow microbiomes of individual whales and identify the presence of potential pathogens. To address these goals, a small, unmanned hexacopter drone (10) (Fig. 1a) was used to collect blow samples from humpback whales (*Megaptera novaeangliae*) from Race Point Channel, north of Cape Cod, MA, and coastal waters surrounding Vancouver Island, in both British Columbia and Washington State. We examined the blow microbiomes, as well as those associated with the surface seawater, by sequencing and comparing partial small-subunit (SSU) rRNA genes of bacteria and archaea. These analyses revealed that blow microbiomes are distinct from seawater and contain an extensive network of consistent core microbiome members whose absence could serve as an important framework for health monitoring.

## RESULTS

### Blow microbiomes are similar between individuals and distinct from surface seawater.

**FIG 2:**
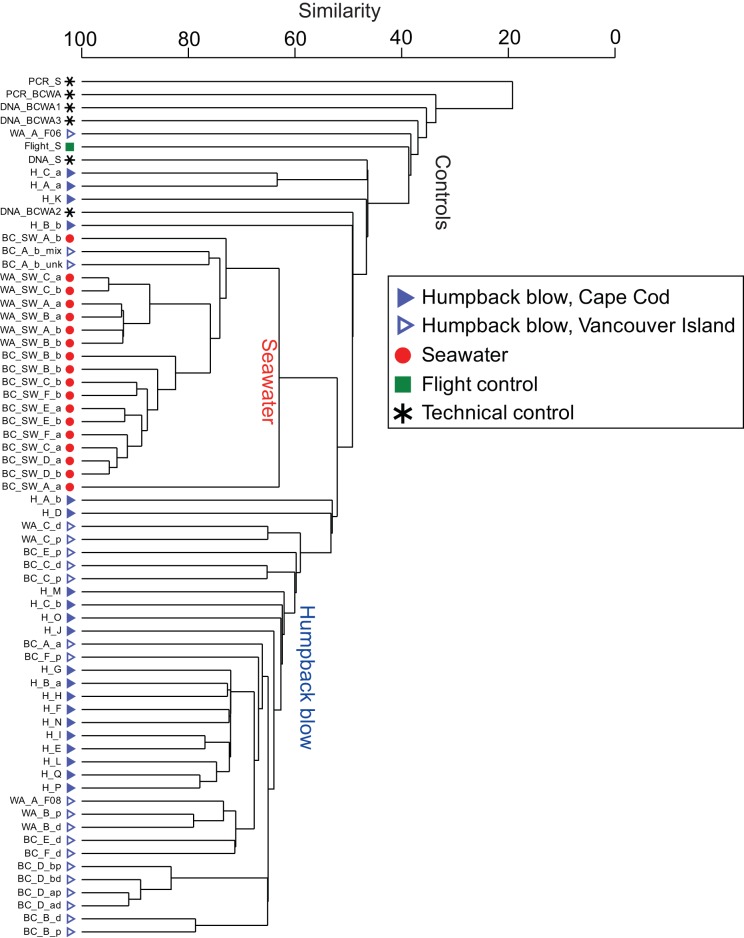
Comparison of humpback blow, surface seawater, and flight and technical control samples using a cluster dendrogram of bacterial and archaeal SSU rRNA genes grouped using minimum entropy decomposition (17) and compared using Bray-Curtis dissimilarity (18). The categories “controls,” “seawater,” and “humpback blow” were inferred based on the clustering patterns and sample types.

By flying a small, remotely operated hexacopter drone 2 to 4 m above humpback whales (Fig. 1b), blow was collected from 17 whales off Cape Cod and nine whales near Vancouver Island (see Table S1 in the supplemental material). Partial bacterial and archaeal SSU rRNA genes were amplified from DNA extractions of the blow samples, including replicate blows from eight animals; environmental controls (a nonblow flight and nine replicate surface seawater samples from around Vancouver Island; sampling equipment was not available for Cape Cod seawater); and technical controls (DNA extraction of sterile swabs and PCR blanks) and sequenced, resulting in 2,337 to 180,141 sequences for the noncontrol samples. Minimum entropy decomposition (MED) (17), a sensitive method for partitioning sequences into operational taxonomic units (here referred to as MEDs or nodes), identified 616 MEDs for the entire data set. A cluster dendrogram analysis of Bray-Curtis dissimilarity (18) of the MEDs showed that community compositions of the technical control samples were similar to each other but different from the majority of blow samples (Fig. 2). Five sparse-volume (volume observed in the field) blow samples (WA_A_F06, H_C_a, H_A_a, H_K, and H_B_b) clustered with the technical controls, indicating that the volume of these samples likely was so low that they only reflected the background microbial signal of technical contaminants such as laboratory reagents. Thus, these five low-volume samples were removed from the data set and are not included in the results presented below. Although two blow samples (BC_A_b_mix and BC_A_B_unk) from the same whale were more similar to the surface seawater than to the other humpback blow samples (Fig. 2), seawater microorganisms may be incorporated into the blow; hence, these blow samples were included in all further analyses. The compositions of the humpback blow microbiotas were significantly different from those of the microbiotas of surface seawater (permutational multivariate analysis of variance [PERMANOVA], *F* = 61.364, *P* < 0.001). Although the compositions of the humpback whale blow microbiotas were 50 to 90% similar to each other, the microbiotas of the blow samples collected off Cape Cod were nevertheless significantly different from those collected around Vancouver Island (PERMANOVA, *F* = 5.8224, *P* < 0.001), and neither finding was impacted by the factor of sequencing depth (PERMANOVA with pairwise tests, *P* > 0.05). The technical controls were used only to assess the microbial signal of blow and seawater samples, and while they appear in Fig. 2, they are not represented in any further analyses but do serve as a constant source for assessing contamination (see below description of core microbiome).

10.1128/mSystems.00119-17.1 TABLE S1  Details of humpback blow, seawater, and flight and technical control samples examined in the study. Download TABLE S1, XLSX file, 0.01 MB. Copyright © 2017 Apprill et al. 2017 Apprill et al. This content is distributed under the terms of the Creative Commons Attribution 4.0 International license.

### Diverse assemblage of microorganisms found in humpback blow.

**FIG 3:**
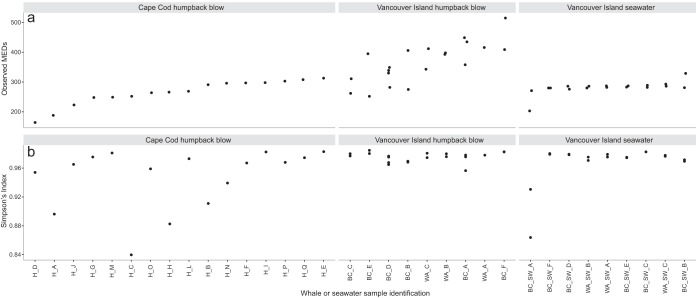
Diversity of whale blow and seawater samples from minimum entropy decomposition (MED) node groupings (17), including observed number of MEDs, a relative estimate of species richness between samples (a), and Simpson’s index (19), a relative estimate of diversity and evenness (b). Data from replicate collections are shown for the Vancouver Island blow and seawater samples with multiple symbols per sample name.

The humpback whale blow samples contained a diverse assemblage of microorganisms, in terms of both richness and phylogeny. In whale blow, the observed number of MEDs, which is comparable to a fine-resolution species richness index, ranged from 164 to 515, with an average of 321 (Fig. 3a). The number of MEDs in surface seawater samples generally fell into this range as well, suggesting that the blow and surface seawater support a similarly rich community of cells (Fig. 3a). There was considerably more consistency between the numbers of observed MEDs within replicate samples in the seawater microbiome than for the Vancouver Island humpback whale blow samples (the only blow samples that were replicated), which could be related to inconsistencies in whale blows, volume of blow collection, or sequencing depth (Fig. 3a). Simpson’s index of diversity (19), which also considers evenness, generally ranged above 0.90, indicating high microbial diversity in the samples. The Simpson index was comparable for blow and seawater samples from Vancouver Island but was more variable for the Cape Cod blow samples (Fig. 3b).

**FIG 4:**
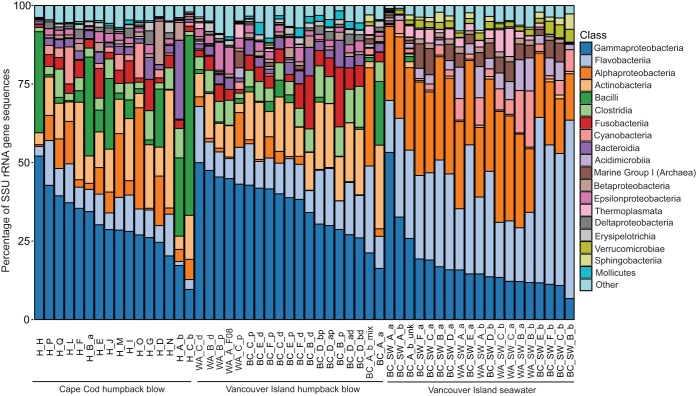
Overview of the phylogeny of the bacteria and archaea associated with the humpback whale blow and seawater samples on a class level based on partial SSU rRNA gene sequences.

A phylogenetically diverse assemblage of sequences that spanned 15 phyla of *Bacteria* and two phyla of *Archaea* was identified in the humpback whale blow microbiomes. Several classes were shared with the seawater samples, including *Gammaproteobacteria*, *Flavobacteriia*, and *Alphaproteobacteria* (Fig. 4). However, the whale blow samples from both locations harbored classes not common in the surface seawater, such as *Actinobacteria*, *Bacilli*, *Clostridia*, *Fusobacteriia*, *Bacteroidia*, *Acidimicrobiia*, *Epsilonproteobacteria*, *Deltaproteobacteria*, *Erysipelotrichia*, and *Mollicutes* (Fig. 4).

### Extensive core microbiome in whale blow with relatedness to other marine mammals.

**FIG 5:**
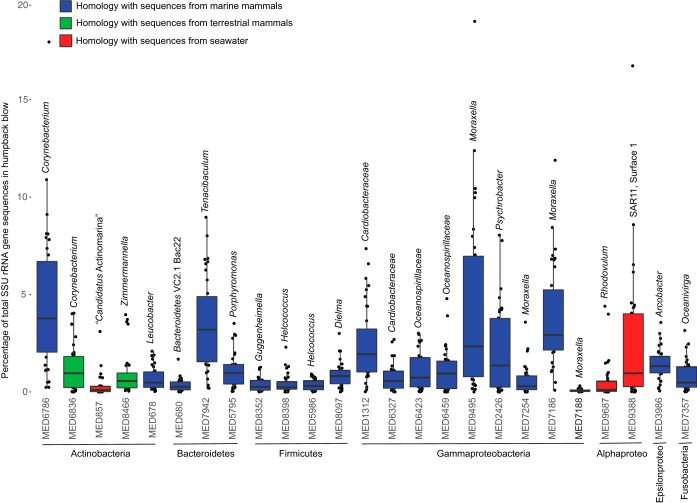
Box plots displaying the median, minimum, maximum, and first and third quartiles of the percentage of the 25 members of the core microbiome detected in all humpback whale blow samples, obtained from minimum entropy decomposition (17) nodes of bacterial and archaeal SSU rRNA genes. The phyla or subphyla (for *Proteobacteria*) of each MED are listed below the box plot, the most detailed level of taxonomy available from the SILVA database (v.123) (52) is shown above, and the colors refer to sequences with homology to those previously recovered from marine mammals (blue), terrestrial mammals (green), or seawater (red). The color of MED 7188 is blue.

Twenty-six MEDs were present in all humpback whale blow samples. However, one of these MEDs, 6038, identified as *Bacillus*, was considered a technical contaminant and not a member of the blow microbiome because it was found in all technical control samples and is a common contaminant in laboratory reagents (20). The remaining 25 MEDs common to all blow samples were considered “core” members of the humpback whale blow microbiome. These core microbiome members spanned seven phyla or classes (for the *Proteobacteria*) and ranged in relative abundance from 0.01 to 18% of the total community in each blow sample (Fig. 5). Collectively, the 25 core microbiome members comprised 36.0% (standard deviation [SD], 10.5%) of the total humpback blow sequences from whales residing in both geographic locations.

Phylogenetic analysis of the core microbiota using ARB and the SILVA database (v.128) revealed that 20 of the 25 core members were most closely related to microbial sequences recovered from other species of marine mammals, most commonly from the mouths and blowholes of bottlenose dolphins (Fig. 5; Table 1). Core members with homology to other marine mammal-associated microbes that were represented at mean abundances of 1% or greater in all humpback blow samples were *Corynebacterium* MED6786 (bottlenose dolphin forestomach), *Tenacibaculum* MED7942 (bottlenose dolphin blowhole), *Porphyromonas* MED5795 (bottlenose dolphin mouth and humpback whale skin), *Cardiobacteriaceae* MED1312 (bottlenose dolphin blowhole), *Oceanospirillaceae* MED6423 and MED6459 (bottlenose dolphin forestomach and blowhole), *Moraxella* MED9495 and MED7186 (bottlenose dolphin mouth), *Psychrobacter* MED2426 (bottlenose dolphin mouth and blowhole), and *Arcobacter* MED3986 (bottlenose dolphin blowhole and forestomach). The nine other core MEDs with homology to sequences recovered from marine mammals were present at mean abundances less than 1% (Fig. 5; Table 1). Additionally, two of the core members, *Corynebacterium* MED6836 and *Zimmermannella* MED8466, shared homology to sequences previously identified in terrestrial mammals (Fig. 5; Table 1). Last, three core members present in the humpback blow shared homology to sequences commonly recovered from seawater: “*Candidatus* Actinomarina” MED857; the *Roseobacter*-affiliated *Rhodovulum* 9687; and SAR11, Surface 1 clade 9833. Indeed, these three seawater-affiliated MEDs were well represented in the seawater samples, each present at average abundances ranging from 4.3 to 5.1%.

**TABLE 1:** List of core MED nodes of SSU rRNA gene sequences with taxonomic affiliations and description of environment where the most similar sequences were recovered

MED	Taxonomic affiliation	Environment of most similar sequences (GenBank identifier)
6786	*Corynebacterium*	Bottlenose dolphin forestomach (JQ192966)
6836	*Corynebacterium*	Horse uterus (CP011546)
857	“*Candidatus* Actinomarina”	Seawater next to bottlenose dolphin (JQ195517)
8466	*Zimmermannella*	Human skin (GQ043066)
678	*Leucobacter*	Bottlenose dolphin blowhole (FJ959933)
680	*Bacteroidetes* VC2.1 Bac22	Bottlenose dolphin mouth (JQ210604)
7942	*Tenacibaculum*	Bottlenose dolphin blowhole (FJ959464)
5795	*Porphyromonas*	Bottlenose dolphin mouth (KC259428) and humpback whale skin (GU202009)
8352	*Guggenheimella*	Bottlenose dolphin mouth (JQ208689)
8398	*Helcococcus*	Bottlenose dolphin mouth (FJ959814)
5986	*Helcococcus*	Sea lion rectum (JQ208548)
8097	*Dielma*	Bottlenose dolphin mouth (JQ209430)
1312	*Cardiobacteriaceae*	Bottlenose dolphin blowhole (FJ960054)
6327	*Cardiobacteriaceae*	Bottlenose dolphin mouth (KC260696)
6423	*Oceanospirillaceae*	Bottlenose dolphin forestomach (JQ194233) and blowhole (FJ959835)
6459	*Oceanospirillaceae*	Bottlenose dolphin forestomach (JQ193528) and blowhole (FJ959835)
9495	*Moraxella*	Bottlenose dolphin mouth (JQ216648)
2426	*Psychrobacter*	Bottlenose dolphin blowhole (FJ960065) and mouth (KC260479)
7254	*Moraxella*	Bottlenose dolphin mouth (JQ216648)
7186	*Moraxella*	Bottlenose dolphin mouth (JQ216648)
7188	*Moraxella*	Bottlenose dolphin mouth (JQ216648)
9687	*Rhodovulum*	Sub-Antarctic seawater (AY697867)
9388	SAR11, Surface 1 clade	Gulf of Mexico seawater (KU578707)
3986	*Arcobacter*	Bottlenose dolphin blowhole (FJ959747) and forestomach (JQ194125)
7357	*Oceanivirga*	Bottlenose dolphin forestomach (JQ193505) and mouth (KC260320)

### No cetacean respiratory pathogens detected in humpback blow.

**FIG 6:**
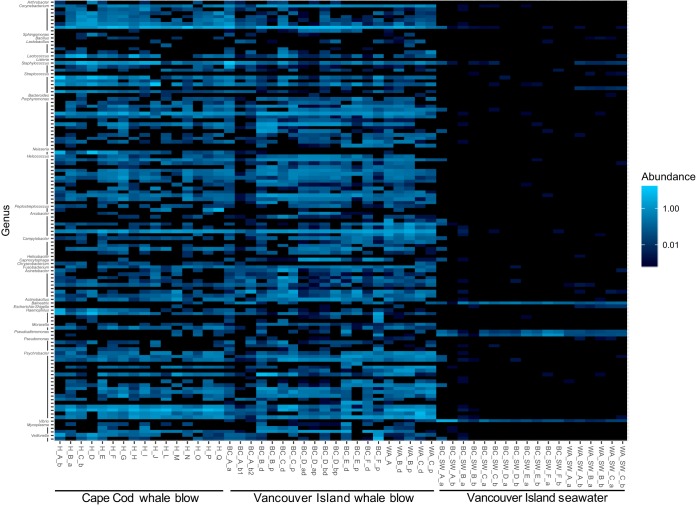
Heat map of the distribution and relative abundance of the minimum entropy decomposition nodes (MEDs) (17), obtained from bacterial and archaeal SSU rRNA genes, from humpback whale blow and surface seawater samples that are related to potential pathogens. The MEDs are classified to genus level, and the line below a genus name indicates membership within the same genus.

To identify samples that might be suitable for respiratory pathogen screening, the taxonomy of the blow MEDs was screened at the level of genus against a custom pathogen database that included human and animal pathogens recognized by the American Biological Safety Association, as well as previously identified and potential pathogens from studies of marine mammals (7, 21–27) (Table S2). One hundred fifteen MEDs from whale blow and seawater spanning 31 genera had a genus-level phylogenetic affiliation with pathogens listed in the database (Fig. 6). The pathogen relatives identified in the whale blow were numerous and distinct from those present in the seawater samples (Fig. 6). Of the potential pathogens identified in the blow samples, 10 genera were previously identified in other species of marine mammals, and of those, *Corynebacterium* was the only genus also represented in the core microbiome of humpback whale blow (Table 2). Specifically, two uncharacterized *Corynebacterium* species were previously cultivated from the spleen, blood, and lymph nodes of bottlenose dolphins following mortality (21), although it should be noted that this genus harbors many nonpathogens that often associate with healthy humans (28, 29). Of the potential pathogens identified in the humpback blow samples, none were known cetacean respiratory pathogens.

10.1128/mSystems.00119-17.2 TABLE S2  Description of potential marine mammal as well as known human, animal, and plant pathogens identified from the American Biological Safety Association (ABSA) risk groups that comprise the custom pathogen database. Download TABLE S2, XLSX file, 0.04 MB. Copyright © 2017 Apprill et al. 2017 Apprill et al. This content is distributed under the terms of the Creative Commons Attribution 4.0 International license.

**TABLE 2:** List of potential pathogens identified in the humpback whale blow according to genus-level identity, as well as descriptions of recognized pathogens and putative marine mammal pathogens

Genus	MED(s) in whale blow^a^	Putative marine mammal pathogen (reference[s])	No. of recognized pathogens in genus
*Acinetobacter*	9761, 8888, 5164, 8857, 5165, 1070, 9765, 1067	*Acinetobacter lwoffii* (21)	NA^b^
*Actinobacillus*	6581	NA	9
*Arcobacter*	8192, **3986**, 8223, 8193, 8194, 3989, 8222	NA	2
*Arthrobacter*	100	*Arthrobacter cumminsii* (16)	NA
*Bacillus*	6010	NA	4
*Bacteroides*	6758	NA	14
*Balneatrix*	4335	NA	1
*Campylobacter*	3645, 3641, 3642, 3651, 80	NA	11
*Capnocytophaga*	6603	NA	6
*Chryseobacterium*	7829	NA	4
*Corynebacterium*	**6786**, 6788, **6836**, 8673, 8671, 6797, 8652, 8654, 3514	*Corynebacterium* spp. (21)	33
*Escherichia-Shigella*	5544	*Escherichia coli* (21)	0
*Fusobacterium*	35	*Fusobacterium varium* (21)	13
*Haemophilus*	9046, 9047, 9045, 5437	NA	18
*Helcococcus*	4258, 8395, **8398**, 8377, 261, 5988, 8396, 1947, 1949, 257, 8397,8378, **5986**, 260	NA	2
*Helicobacter*	3988	NA	12
*Lactobacillus*	4629, 4625, 4645, 322	NA	3
*Lactococcus*	2216	NA	1
*Listeria*	5860	NA	2
*Moraxella*	7227, 8609	NA	9
*Mycoplasma*	7640, 3008, 7642	*Mycoplasma* sp. (16)	63
*Neisseria*	9019, 9020	NA	11
*Peptostreptococcus*	8129, 8130	NA	10
*Porphyromonas*	9273, 9078, 9274, 5800, 9077, 9112, 5796, 9114, 5799, **5795**, 5797, 9109, 9080, 5486	NA	12
*Pseudoalteromonas*	5116, 5119	NA	1
*Pseudomonas*	9740, 5159, 9739, 5873	*Pseudomonas aeruginosa* (7)	9
*Psychrobacter*	8626, 2428, 2426, 7254, 2065, 8585, 4878, 8627, 8584, 2062, 2441,4879, 7255, 8610, 9480, 9482, 8582, 7157, 4874	NA	1
*Sphingomonas*	231	NA	2
*Staphylococcus*	6011, 6041, 6038	*Staphylococcus aureus* (7, 16), *Staphylococcus cohnii* (16), *Staphylococcus epidermidis* (16, 21), *Staphylococcus warneri* (16), *Staphylococcus* sp. (16), *Staphylococcus delphini* (23)	13
*Streptococcus*	7098, 3720, 7097, 2227, 2217, 2228	Alpha-hemolytic *Streptococcus* (16), *Streptococcus zooepidemicus* (7), *Streptococcus* group D (21)	27
*Veillonella*	1666, 1668	NA	1
*Vibrio*	2695	*Vibrio anguillarum* (16), *Vibrio alginolyticus* (16), *Vibrio wodanis* (16), *Vibrio* sp. (21)	15

^a^ MED nodes (17) present in the core microbiome are in bold.

^b^ NA, not applicable.

## DISCUSSION

In the first examination of whale blow microbial diversity, we show that blow from humpback whales supports a diverse and rich community of microorganisms with a number of features that may be useful for monitoring health. First, our study demonstrates that the microbial communities in blow and surface seawater are different, indicating that whale blow is not just aerosolized seawater. Although both baleen and toothed whale blows were previously compared to seawater (10, 16), this was the first study to apply more comprehensive microbial diversity analyses to whale blow. Indeed, there were some surface seawater-associated bacteria within the humpback blow examined in our study, but this is not surprising because seawater may remain in the upper tract between breaths and enter and leave the blowhole cavity and the upper nasal tract of a surfacing whale during the inhalation phase (M. Moore, unpublished observations), to the point where these samples could be described as seawater lavages of the upper respiratory tract seeded with condensed exhalation. Three humpback blow core MEDs, including bacteria from the globally abundant SAR11 clade (30), were well represented in the seawater samples, at relative abundances of 4 to 5%, suggesting that the most abundant cells in the seawater are likely the cells making their way into the upper respiratory tract. Furthermore, the remaining 22 core microbiome sequences were most closely related to marine and terrestrial mammals, suggesting that these cells were, indeed, coming from the whales, not the seawater. The exhaled breath of most mammals is believed to comprise cells that originate from both the mouth and the nasopharynx because they are anatomically connected (31–33). Because the nasopharynx of cetaceans is not connected to the mouth, cetacean breath passes through only the respiratory tract; therefore, the breath microbes originating from the cetacean are from the respiratory tract and almost certainly do not include oral microbes. Two core members of the blow microbiome were previously detected on humpback whale skin, *Porphyromonas* (MED5795) and *Psychrobacter* (MED2426) (34). It is possible that these whale skin-associated microbes reside on the epithelium of the blowhole and become aerosolized with the force of the whale’s exhalation. Thus, in addition to seawater-associated microbes, a mixture of pulmonary bacteria and microbes associated with the epithelial cells of the blowholes likely comprise the blow microbiome in cetaceans.

The second and possibly most useful feature of the humpback whale blow microbiome for health monitoring is that it contains a surprisingly high number of core microbiome members shared by all individuals, despite our samples being collected from different populations residing in distinct ocean basins. Defining core microorganisms in a host environment is helpful because the persistence of core members between individual animals suggests that these core microbes may be beneficial for the host (35, 36). Core microbiomes are generally identified by applying criteria such as sequences or taxonomic groups that are present in 30 to 80% of hosts, and only rarely is 100% membership within all hosts used as the defining criterion (35). Here, we used the 100% host membership criterion at a highly discriminative species-type level of phylogenetic similarity (as determined by MED) and identified 25 core microbiome members, which to our knowledge is an unprecedented number of core members at this discriminative scale for any marine or terrestrial host microbiome. The most similar finding using the 100% core membership criterion is for the human gut and hands, which host 18 and 5 core microbial members, respectively (37, 38). While core membership in the human respiratory tract has not been investigated extensively, studies suggest high variability in the lower respiratory tract microbiome between individuals (39). Stable and persistent core microbiomes with low interindividual variability, as observed here for the humpback whales, suggest that the microbiome and/or host may receive benefits from the presence of this collective group of cells, such as nutritional or immune benefits. However, examining blow microbiomes from unhealthy or diseased animals will be necessary to understand if the core microbiome does indeed change with health state. Blow samples from populations of large whales with healthy and unhealthy individuals, such as the North Atlantic right whales, may be particularly useful for this comparison (40).

Another reason that the presence of a core microbiome in whale blow could be a useful feature for monitoring health is that the absence of core members and/or the presence of atypical microbes in the blow could suggest an alteration in the growth environment or immune response of the microbes, as is typical for pulmonary infections and diseases (33). Analyzing microbiomes associated with blow from humpback whales with known pulmonary conditions would greatly advance our understanding about the stability of the core microbiome. However, these conditions are challenging to diagnose in the wild. Instead, examining the blow microbiomes of whales with field-diagnosable conditions, such as poor body condition detectable by drone-enabled photogrammetry (41), will help advance our understanding of the variability of the core microbiome in relation to health.

The third feature of the blow microbiome of humpback whales that is important for health monitoring is that the microbiomes can be coarsely screened for pathogens using the amplicon sequencing approach. We chose to screen for relatives of pathogens, instead of just species-level identity to known pathogens, because cetacean pathogens are not well described, and the pulmonary and blowhole system of cetaceans is unique compared to other mammals. As knowledge of specific cetacean respiratory pathogens increases, a stricter sequence similarity approach could be applied. Until that time, analyzing blow microbiomes for potential pathogens using amplicon sequencing provides a means to screen for samples for further analysis of pathogens using quantitative PCR (qPCR) or metagenomics. Additionally, this amplicon-based method provides a means to understand the other possibly mutualistic or commensal members of the respiratory microbiome that would not be available if analysis were limited to pathogen detection. In this study, no known marine mammal respiratory pathogens were identified for further verification using qPCR or metagenomics. Recently, Raverty et al. suggested that potentially pathogenic bacteria and fungi, including some exhibiting resistance to antimicrobial agents, identified in killer whale blow and surface seawater may pose a threat to the endangered killer whale population (16). Large whales, including humpbacks, frequently reside in coastal areas of high anthropogenic influence, including wastewater and sewage dispersal (42, 43), and therefore, the probability of whales being exposed to pathogens is high. Whales also exhibit behaviors, such as surfacing in close proximity to each other or feeding cooperatively (44, 45), which could facilitate the spread of pathogens between individuals. Thus, screening for potential pathogens in the blow microbiome is a particularly useful endeavor for large whales.

A fourth useful feature of the blow microbiome is that the richness, diversity, and sequence homology of microorganisms detected in whale blow were on par with those previously identified in the blowholes of bottlenose dolphins (13–15), the only comparable microbiome data sets (i.e., broad taxonomic microbiome surveys that used cultivation-independent approaches). This feature suggests that blow microbiome monitoring criteria for one species may be applicable to other cetaceans. Although the criteria for sequence delineation differed between this study (which used MEDs) and the previous dolphin studies (which used 97% sequence similarity), all studies examined surface seawater and found similar microbiome richness patterns between the marine mammal and seawater environments, thus making the data sets comparable on this generalized level.

To further advance the blow microbiome as a health monitoring tool for large whales, several features require consideration and future development. While drones are safer and less invasive to the whale than pole sampling, they can result in low-volume samples. Indeed, several low-volume samples collected from this study were found to be similar in composition to samples from flight and technical controls. Contaminating DNA is frequently identified in low-volume samples (20, 46), including those targeting low microbial biomass from human lungs (39), and the present study suggests that flight and technical controls are necessary to ensure that biological samples have sufficient volume and microbial biomass for accurate comparisons. Also, the differences in richness and community composition that were observed in the replicate blow samples were probably related to inconsistencies in whale blows or blow collections and signify the importance of repeated sampling. In addition to these recommendations, we acknowledge that this study presents a limited description of the microbiome by focusing on bacteria and archaea. Protists, viruses, and fungi can also be important indicators of respiratory illnesses (26, 47), and detection of these organisms may be achievable on some of the higher-volume samples using a metagenomics-based sequencing approach. Efforts should also be made to examine the growth environment for the blow-associated microorganisms, including characterization of blow chemistry, temperature, and salinity, which could inform conditions for microbial cell growth, enhance cultivation efforts, and ultimately allow us to better evaluate the beneficial, commensal, or pathogenic potential of the microbial cells residing in the whale’s respiratory tract. Last, this and a previous study (10) used drones to collect blow-associated microorganisms from large whales, where this approach appears to be broadly applicable. These methods may also be applicable to smaller cetaceans, but this still needs to be examined in the context of the ability of small unmanned aerial systems (UAS) to operate near small cetaceans unobtrusively.

### Conclusions.

This study demonstrates that remote sampling via drones provides a noninvasive means to collect whale blow for microbiome analysis. Using this technique, we showed that different humpback whales and populations harbor similar microbial communities in their exhaled blow, including the presence of a large number of specific core bacteria shared between all individuals. The persistence of these core members in apparently healthy individuals suggests that they may be indicative of a healthy, noninfected pulmonary system, and their presence or absence could be informative for health monitoring of humpback whales and possibly other large whales.

## MATERIALS AND METHODS

### Sample collection.

Exhaled breath condensate (blow) was collected from humpback whales (*Megaptera novaeangliae*) in the Race Point Channel, north of Cape Cod, MA (*n* = 17), during July 2015 and in two locations around Vancouver Island: Johnstone Strait and surrounding channels, British Columbia (*n* = 6 whales with replicate samples for all animals), in August 2016 and off San Juan Island, Washington State (*n* = 3 whales with replicate samples for 2 animals), in September 2016. Samples were collected using a small, unmanned hexacopter drone (APH-22; Aerial Imaging Solutions, Old Lyme, CT) operated by a pilot and copilot team from a vessel (48) (Fig. 1). Collection surfaces differed between the flights and locations. For Cape Cod, the hexacopter’s dome and one to three 96-well PCR plates fixed to the struts of the hexacopter were used as the sterile collection surfaces. For Vancouver Island, one forward-facing sterile, 96-well PCR plate; the dome of the hexacopter; and, for some whales, a 150-mm-diameter sterile petri dish affixed to the top of the dome (Fig. 1a) were used. Prior to flight, the hexacopter’s propellers, arms, struts, and dome were sterilized with 95% isopropanol. The hexacopter then was flown 2 to 4 m or more above the blowhole, and once the whale exhaled, the hexacopter was returned to the boat so that the sample could be processed. Using sterile technique, the blow was swabbed from the collection surfaces using sterile cotton-tipped swabs or flocked swabs (Copan Diagnostics, Inc., Murrieta, CA). Each swab then was placed in a sterile 2-ml cryovial, frozen in a liquid nitrogen vapor shipper, and transferred to −80°C until processing. In one case, a sample had a large-enough volume to pipette and thus was pipetted directly into a 2-ml cryovial prior to freezing. Sampled whales were photographed for identification purposes using standard methods to identify duplicate samples from the same whale (49).

As a control, the hexacopter was flown with sterile PCR plates attached for the same flight duration, altitude over the water, and distance from the boat as had been done when collecting actual blow samples. Upon landing, a sterile cotton-tipped swab was used to wipe the PCR plates and was placed in a sterile 2-ml cryovial, frozen, and stored in the same manner as the blow samples. To sample surface seawater microbes, 1 liter surface seawater was collected at 0.25-m depth from the same general area as the blow collections around Vancouver Island and filtered through an 0.22-μm Supor membrane filter (Millipore, Boston, MA) using a peristaltic pump. Each filter was placed in a sterile 2-ml cryovial, frozen in a liquid nitrogen vapor shipper, and transferred to −80°C until processing. Two replicate seawater samples were collected per sampling site.

### Sample preparation, PCR amplification, and sequencing.

Nucleic acids were isolated from the swabs, 50 µl of the pipetted sample, or the filters using the PowerBiofilm DNA isolation kit (Mo Bio Laboratories, Inc., Carlsbad, CA). Barcoded 515FY and 806RB (50, 57) primers, utilized by the Earth Microbiome Project, were used to amplify the V4 region of the SSU rRNA gene in triplicate 25-µl PCR mixtures per sample on a Bio-Rad Thermocycler (Hercules, CA) as follows: an initial denaturation step at 95°C for 2 min; 30 to 38 cycles (blow samples) or 20 to 25 cycles (seawater samples) of 95°C for 20 s, 55°C for 15 s, and 72°C for 5 min; and an extension step at 72°C for 10 min. Each PCR mixture contained 1 ng of DNA, 200 nM barcoded primers, Go*Taq* Flexi DNA polymerase, Go*Taq* Flexi 5× colorless buffer, 2.5 mM MgCl_2_, and 200 µM deoxynucleoside triphosphate (dNTP) mix (Promega, Madison, WI). Products of the triplicate reactions were combined for each sample, screened on a 1% agarose–Tris-borate-EDTA (TBE) gel using HyperLadder (50 bp; Bioline USA Inc., Taunton, MA) to confirm amplicon size, and purified either with a 1.5% agarose-TBE gel using the MinElute gel extraction kit (Qiagen, Valencia, CA) or without the gel extraction using a Wizard PCR cleanup system (Promega, Madison, WI). A low-concentration microbial mock community with equimolar rRNA operon counts (obtained through BEI Resources, NIAID, NIH, as part of the Human Microbiome Project; genomic DNA from microbial mock community B [even, low concentration], v5.1L, for 16S rRNA gene sequencing, HM-782D) was amplified and sequenced with the samples to test for sequencing errors. To test for reagent contamination from the DNA isolation kit, 50 µl sterile water or a dry swab was included in each batch of isolations. Each PCR run included sterile water as a negative control, for which amplification was not detected via gel electrophoresis for any of the runs. After quantification on a Qubit 2.0 fluorometer with a double-stranded DNA (dsDNA) high-sensitivity assay kit (Invitrogen Corp., Carlsbad, CA), the PCR products were pooled into two libraries of equal concentrations. Amplicons were sequenced over two 2- by 250-bp MiSeq (Vancouver Island) and NanoSeq (Cape Cod) formats (Illumina, San Diego, CA) at the University of Illinois W. M. Keck Center for Comparative and Functional Genomics.

### Sequence data processing.

Raw sequences (6,358,878) were assembled, denoised, and quality filtered using mothur v.1.36.1 (51). Specifically, barcoded primers were removed, sequence reads were joined, sequences were trimmed to 255 bp, and ambiguous base pair calls were removed, resulting in sequences with an average of 253 bp. The sequencing error rate was calculated as 0.0015771% from the mock community samples. Sequences from technical and flight control samples did not meet quality control criteria. However, due to the low-volume nature of the blow samples, these samples were initially included in the analysis (Fig. 2). Sequences were classified using a k-nearest neighbor consensus algorithm in mothur with the SILVA rRNA sequence database (v.123) (52, 53), and those that were identified as chloroplasts (233,253 sequences, from the seawater samples) and unknown (355 sequences) were removed from the data set. Chimeric sequences found by UCHIME (54) within mothur also were removed, and the cumulative outcome of these quality control measures resulted in 4,903,825 sequences (22.9% loss of sequences). Minimum entropy decomposition (MED) (17) was used to bin sequences to homogeneous operational taxonomic units (here referred to as MEDs) on the entire data set, 4,903,825 sequences, with a minimum substantive abundance (*M*) set to 490 to reduce the impact of noise, resulting in 616 nodes and a further reduction of the data to 4,183,042 reads. Taxonomy was assigned to sequences representing each MED using the k-nearest neighbor consensus algorithm in mothur with the nonredundant SILVA rRNA sequence database v.123 that was customized for humpback whale skin for an unrelated study by adding *Tenacibaculum* and *Psychrobacter* hypervariable region IV sequences from the partial (shorter-read) version of the same database.

### Microbial community analysis.

Using the Primer software (v7.0.9; Primer-E, Auckland, New Zealand), Bray-Curtis dissimilarity (18) was calculated from nonrarefied, square-root-transformed relative abundances of the MED nodes and compared using a single linkage clustering algorithm. The resulting dendrogram showed that five sparse-volume blow samples clustered with the technical control samples (DNA isolation, PCR, and mock community controls). Based on this high similarity to the technical controls, these samples were considered to have been of such low volume that they only reflected the background microbial signal of the lab reagents. Therefore, these five samples were removed from the remaining analyses. Differences in microbial community compositions between whale populations and between seawater and whale blow were tested on Bray-Curtis dissimilarity (18) using PERMANOVA+ (v7.0.9; Primer-E, Auckland, New Zealand) with replicate samples treated as a random effect and sequencing depth also tested as a factor using the categories of <50,000 and >50,000 sequences. The phyloseq package (55) in R was used to examine alpha diversity of the humpback blow and seawater MEDs, without repeated subsampling, including richness and Simpson’s index of diversity (19). R also was used to determine the core MEDs (MEDs present in all high-quality samples of humpback whale blow). Taxonomic affiliations of the core microbiome and most-homologous sequence were determined using ARB with the most recent nonredundant SILVA database (v.128) at the time of analysis. The ggplot2 package (56) in R was used to construct the taxonomic stacked bar and box plot figures.

### Pathogen database and screening.

A custom pathogen database of phylogenetic affiliations was constructed to screen the humpback whale blow microbiome sequences for the presence of potential pathogens at the genus level. The database included putative pathogens of any marine mammal body site identified from a compilation made by Raverty and colleagues (16) and from other published studies (see Table S2 in the supplemental material). To account for any bacteria that have not yet been identified as marine mammal pathogens, the database also included any human and animal bacterial pathogens recognized by the American Biological Safety Association (899 pathogens). To ensure that the humpback blow and seawater MEDs were resolved to the most descriptive taxonomic level offered by the SILVA database, the representative sequences were assigned taxonomic identity using both the k-nearest neighbor algorithm in mothur with the SILVA rRNA sequence database customized for humpback whale skin as mentioned above and the SINA Alignment Service v1.2.11 (52).

### Data availability.

Sequence data from this study are available at NCBI under BioProject accession no. PRJNA401637. Representative MED sequences are available in fasta format in Data Set S1 in the supplemental material.

10.1128/mSystems.00119-17.3 DATA SET S1  Representative SSU rRNA gene sequences of the minimum entropy decomposition nodes. Download DATA SET S1, TXT file, 0.2 MB. Copyright © 2017 Apprill et al. 2017 Apprill et al. This content is distributed under the terms of the Creative Commons Attribution 4.0 International license.
